# Hypolipidemic Effects of Soy Protein and Isoflavones in the Prevention of Non-Alcoholic Fatty Liver Disease- A Review

**DOI:** 10.1007/s11130-022-00984-1

**Published:** 2022-06-09

**Authors:** Chao-Wu Xiao, Amy Hendry

**Affiliations:** 1 Nutrition Research Division, Bureau of Nutritional Sciences, Food Directorate, Health Products and Food Branch, Health Canada, 2203C Banting Research Centre, Ottawa, ON K1A 0L2 Canada; 2 Food and Nutrition Science Program, Department of Chemistry, Carleton University, 1125 Colonel By Drive, Ottawa, ON K1S 5B6 Canada

**Keywords:** Soy, Isoflavones, Hypolipidemic, Non-alcoholic fatty liver disease, Human and animal studies

## Abstract

Non-alcoholic fatty liver disease (NAFLD) is the most common chronic liver disease and affects about 25% of the population globally. Obesity and diabetes are the main causes of the disease characterized by excessive accumulation of lipids in the liver. There is currently no direct pharmacological treatments for NAFLD. Dietary intervention and lifestyle modification are the key strategies in the prevention and treatment of the disease. Soy consumption is associated with many health benefits such as decreased incidence of coronary heart disease, type-2 diabetes, atherosclerosis and obesity. The hypolipidemic functions of soy components have been shown in both animal studies and human clinical trials. Dietary soy proteins and associated isoflavones suppressed the formation and accumulation of lipid droplets in the liver and improved NAFLD-associated metabolic syndrome. The molecular mechanism(s) underlying the effects of soy components are mainly through modulation of transcription factors, sterol regulatory element-binding protein-1 and peroxisome proliferator-activated receptor-γ2, and expressions of their target genes involved in lipogenesis and lipolysis as well as lipid droplet-promoting protein, fat-specific protein-27. Inclusion of appropriate amounts of soy protein and isoflavones in the diets might be a useful approach to decrease the prevalence of NAFLD and mitigate disease burden.

## Introduction

Soy foods have been consumed for centuries in Asian countries. Soy protein is one of the major sources of plant-based protein for human consumption. In addition to its nutritional roles as a rich source of indispensable amino acids, soy protein and its associated isoflavones have been extensively studied for their functional properties in various aspects.

Epidemiological investigations have shown that consumption of soy foods is associated with various health benefits such as lower incidences of coronary heart disease and associated mortality [1], obesity, type-2 diabetes, and atherosclerosis [2–4]. Soy protein and isoflavones have been shown to play major roles in modulation of lipid and glucose metabolism in human clinical trials [5, 6], animal studies [7, 8] and cultured cells [9]. Inclusion of soy protein or soy isoflavones in the diet improved hepatic and blood lipid profiles by reducing triglycerides, total and low density lipoprotein (LDL) cholesterol levels and increasing the ratio of high density lipoprotein (HDL)/LDL cholesterol in both human [6,10] and animal studies [2,11,12]. As a result of these benefits, health claims associated with hypocholesterolemic effects of soy protein have been approved in a dozen countries [13].

Increasing evidence has shown that soy intake had beneficial effects in patients with non-alcoholic fatty liver disease (NAFLD) [14–18], and reduced the formation and accumulation of hepatic lipid droplets and ameliorated liver steatosis in animal models of NAFLD [19–22]. Dietary soy improved glucose and lipid metabolism via modulation of insulin secretion and sensitivity in diabetic animal models with NAFLD [7, 8, 23]. NAFLD prevalence is rapidly increasing worldwide, especially in developed countries. Direct pharmacological treatments for NAFLD are not available. Dietary intervention and lifestyle changes are the major strategies for prevention and treatment of NAFLD. This review focuses on the effects of soy proteins and associated isoflavones on the metabolic syndrome of NAFLD in both human and animal studies and current understanding of the molecular event(s) involved in the hypolipidemic actions and NAFLD prevention of soy components.

### NAFLD

NAFLD is the most common chronic liver disease [24]. The typical features of NAFLD are accumulation of high levels of lipids in hepatocytes, usually greater than 5% of liver weight [25], and formation of excessive hepatic lipid droplets [26]. With the progression of NAFLD, histological changes in the liver can range from hepatic steatosis to steatohepatitis, fibrosis, cirrhosis and even hepatocellular carcinoma. Obesity and diabetes are the main causes of the diseases. NAFLD is associated with metabolic syndrome and changes in biomarkers such as increased insulin resistance, hypertension, hyperlipidemia, elevated oxidative stress and increased plasma fibrinogen, alanine aminotransferase (ALT) and aspartate aminotransferase (AST) levels [16].

The global prevalence of NAFLD is approximately 25% [24,27], and affecting about one-third of the population in Western countries [28]. Around 80% of the obese population and 50% of individuals with diabetes have fatty livers [29]. It is predicted that the incidences of NAFLD, non-alcoholic steatohepatitis (NASH), decompensated cirrhosis, hepatocellular carcinoma and liver deaths in different countries or regions will rise by up to 48, 96, 273, 199 and 295%, respectively, by 2030 (Table 1) [30–35]. Thus, strategies to slow the increase in NAFLD prevalence and therapeutic options are urgently needed to mitigate disease burden [30]. Increasing evidence suggests that dietary intervention and lifestyle changes might play a role in preventing and managing NAFLD [36, 37].

**Table 1:** Projected burden of non-alcoholic fatty liver disease (NAFLD) and non-alcoholic steatohepatictis (NASH) by the year of 2030 in different countries and regions based on modelling studies

	NAFLD		Percentage increase by the year of 2030 (%)					
Country or region	Year	Overall rate (%)	NAFLD	NASH	D. cirrhosis*	HCC*	Liver death	References
Australia	2019	22	25	40	85	75	90	[32]
Canada	2019	21	20	35	97	80	107	[33]
Hong Kong	2019	22	11	20	66	67	74	[34]
Saudi Arabia	2017	26	48	96	273	199	295	[31]
Singapore	2019	26	20	36	108	78	113	[34]
South Korea	2019	21	6	21	87	79	92	[34]
Switzerland	2018	24	18	35	52	46	41	[35]
Taiwan	2019	22	8	24	108	84	106	[34]
United Arab Emirates	2017	25	46	87	241	178	27	[31]
United States	2015	34	21	63	168	137	178	[30]

*D. cirrhosis, Decompensated cirrhosis; HCC, hepatocellualr carcinoma

### Major Nutrient Composition of Soybean

Soybean (*Glycine max*) is one of major dietary sources of plant-based protein and soy protein contains all essential amino acids required by human body, which makes soy products almost equivalent to the foods of animal sources in protein quality [38]. For example, the protein digestibility-corrected amino acid scores, a measurement of protein quality, for soy, beef, cow’s milk, and egg are 1, 0.92, 1, and 1, respectively [39–41].

Dry soybean contains 40% protein, 22% fat, 33% carbohydrate including 10.2% dietary fiber, 5% minerals and vitamins [42]. Soy protein is mainly comprised of two storage globulins, 7S β-conglycinin and 11S glycinin [43] (Table 2). *β*-conglycinin has *α*’, *α*, and *β* subunits, and accounting for ~25% of the total protein, while glycinin has acidic (A) and basic (B) polypeptides to form 5 subunits, A1aB2, A2B1a, A3B4, A1bB1b and A5A4B3, and accounting for ~40% of the total protein. The other minor proteins include 2S, 9S, and 15S storage proteins, lectin, Kunitz and Bowman-Birk protease inhibitors, *β*-amylases, lipoxygenases, and urease [43]. Depending on processing, the protein content in soy foods/products can reach over 90% as in the soy protein isolate (SPI) that is usually used in soy-based infant formulas.

**Table 2:** Nutrient content of dry soybean seeds (*per* 100 g dry weight)

Nutrients		Content
Protein (g)		40
*β*-conglycinin (7S) (α’, α, β subunits)		
Glycinin (11S) [Acidic (A), Basic (B) polypeptides]		
Other minor proteins		
2S, 9S, 15S storage proteins		
Lectin, *β*-amylases, lipoxygenases, urease		
Kunitz and Bowman-Birk protease inhibitors		
Fat (g)		22
Carbohydrate (g)		33
Dietary fiber		10.2
Sugars		8
Minerals and vitamins (g)		5
Isoflavones (mg/g protein)		3–5.1
Genistin:Daidzin:Glycitein ≈ 1:1:0.1		

References [42–44]

Isoflavones are one of the most studied bioactive compounds in soybeans, and are closely associated with proteins. Soy foods and soy-based infant formulas are rich sources of isoflavones, and contain about 3–5.1 mg/g protein [44]. Isoflavones are the major soy phytoestrogens, including genistin, daidzin and glycitein. Both genistin and daidzin are conjugated to sugars and present as glycosides in soybeans and most of the soy foods in Western diets. Glycoside isoflavones cannot be absorbed in the body unless hydrolyzed and converted to aglycones, genistein and daidzein by intestinal microflora or *in vitro* fermentation [45]. In addition, Daidzin or daidzein can be metabolized to equol by certain strains of intestinal microflora in the gastrointestinal tract. However, only 30–50% of the adult population can produce and excrete equol in the urine after daily ingestion of soy foods [46].

### Effects of Soy Intake on NAFLD

The hypolipidemic properties of soy components have been shown in human and animal studies as well as in cultured cells [3, 19, 47, 48], and are critical in reducing the risk for certain chronic diseases. The potential impact of soy intake on metabolic syndrome and biomarkers of NAFLD have been investigated in both human and different animal models of NAFLD. The results suggest that soy protein and associated isoflavones might be promising dietary supplements for prevention or treatment of NAFLD.

### Human Clinical Trials

Inclusion of soy protein in diets may improve metabolic syndrome and risk factors associated with NAFLD. Consumption of 30 g soy nuts that contain 11.3 g protein and 102 mg total isoflavones in replacement of the same amount of red meat for eight weeks significantly lowered blood markers for NAFLD including ALT and AST, malondialdehyde (MDA) and fibrinogen levels compared to other non-soy groups in patients with NAFLD (*n* = 45) [18]. Moreover, the fasting blood sugar, serum insulin, high-sensitivity C-reactive protein (hs-CRP) levels, and systolic and diastolic blood pressure in the soy group were lower than in the non-soy groups [17]. The study was conducted in the patients with NAFLD and no other specific disorders, which should have good external validity. All patients completed the study and detailed data of their dietary intake was collected. The weaknesses of this parallel clinical trial include that the adherence of the patients to the designed diets could only be assessed through patients’ self-reported food records instead of measuring plasma or urine isoflavone levels [18]. In another parallel randomized clinical trial, daily drinking of 240 mL soy milk as a part of low-calorie diet for eight weeks significantly reduced serum ALT, hs-CRP [14], and insulin, and improved insulin resistance, and systolic and diastolic blood pressure in the NAFLD patients (*n* = 70) [16]. However, no changes were observed in fatty liver grade and other liver enzymes including AST, alkaline phosphatase, γ-glutamyl transferase, as well as lipid profile and anthropometric indices. The limitations of this study include that the types of dietary interventions were not blinding, and that the interpretations of ultrasound imaging for evaluation of liver steatosis were subjective, and that serum or urine isoflavones could not be determined as markers for the adherence to the intervention, and the study duration was relatively short [14].

The patients with NASH (*n* = 22, 9 women and 13 men) taking meal replacements containing 44% soy protein and 9% milk protein for 24 weeks had significant reduction in body weight, body mass index (BMI), body and liver fat content, serum ALT and AST, and improved glycemic control and lipid profile. The decrease in ALT was strongly correlated with the reduction in abdominal fat, subcutaneous fat, internal fat and AST. One of the weaknesses of the study was the use of ^1^H-magnetic resonance imaging analysis method in the quantification of hepatic steatosis that was not able to assess inflammation or fibrosis. Additionally, the specific effect of soy on the composition of liver fat could not be differentiated from the impact of caloric restriction. Small sample size limited further analysis of the participant subgroups (*i.e*., sex and age groups) [15]. In a randomized double-blind controlled trial, patients with NAFLD received either a daily supplement of 250 mg genistein (*n* = 41) or placebo (*n* = 41) for 8 weeks. The genistein group had significantly lower levels of serum insulin, MDA, TNF-*α*, IL-6, and improved insulin resistance, as well as reduced waist to hip ratio, body fat percentage and triglyceride compared to the placebo. However, BMI, fasting blood glucose, ALT and AST were not different between the two groups [49].

Akahane et al. [50] recently reported that progression of NAFLD and NASH was strongly associated with the production of equol. A clinical study conducted in 38 NAFLD patients (13 men and 25 women) showed that the degree of hepatic fibrosis and ballooning was markedly higher in the equol non-producers than in the producers in women. The percentage of non-producers (*n* = 8) with NAFLD activity score (NAS, including four histological features: steatosis, lobular inflammation, hepatocellular ballooning, and fibrosis) ≥5 was significantly higher than that of the producers (*n* = 17) in women. However, these associations were not observed in men. Limitations of the study include small sample size, a single-center study, unknown exact amounts of soy products consumed, liver fibrosis assessed by less ideal method, and inconclusive causal relationship [50]. The sex-dependent effect of equol may be attributed to the difference in the endogenous estrogen levels, abundance of hepatic estrogen receptor (ER) [51] and responsiveness to equol between males and females. Like the other soy isoflavones, equol is estrogenic and can bind both ERα and ERβ [52]. It was shown that the female liver has higher ER concentration than the male liver [51], and that female liver is more responsive to estrogen exposure than the male liver due to the more efficient nuclear uptake of cytosolic receptor ligand complexes in females than in males [53].

### Animal Studies

**Fig. 1:**
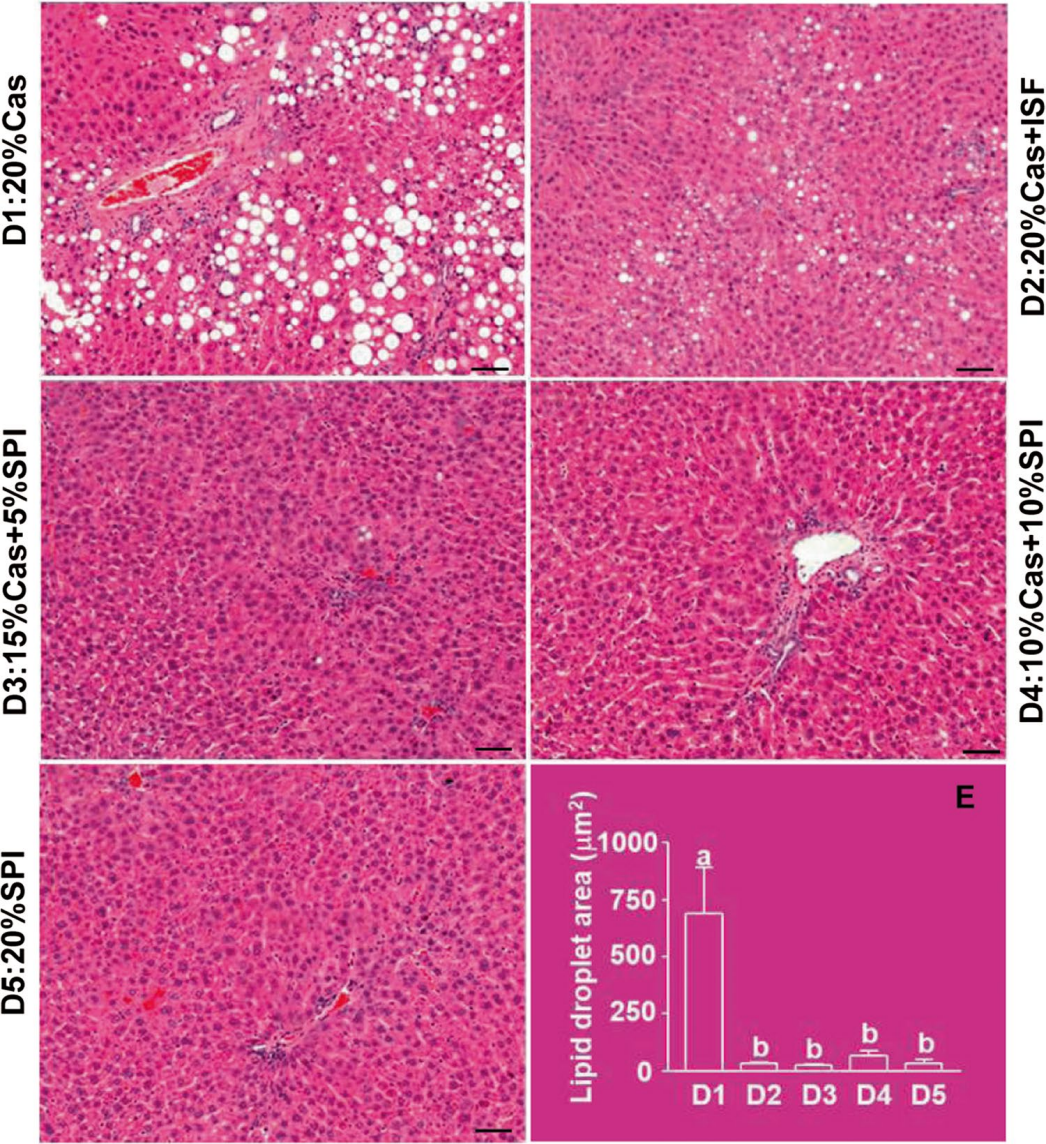
Liver histology of the female Sprague Dawley rats fed diets containing 20% casein in the absence (D1) or presence (D2) of supplemental isoflavones (ISF, 50 mg/kg diet) or increasing amounts of alcohol-washed soy protein isolate (SPI), 5% (D3), 10% (D4) or 20% (D5) in replacement of the same amounts of casein for 90 days. For the assessment of hepatic lipid droplet (HLD) formation and accumulation, the sections were stained with hematoxylin and eosin. The circumference of 100 randomly selected fat droplets in five fields of each section at 20× was measured under microscope using the software Northern eclipse version 7.0. The scale bars represent 10 *μ*m, and the total areas of HLD were presented (E), and the means in (E) with different letters (a, b) differ. (Adapted and reformatted from Xiao et al. [55])

The hypolipidemic functions and preventive effects of soy on NAFLD have also been shown in genetically obese or high-fat/high-sugar induced obese rodent models. Feeding soy protein-containing diet attenuated hepatic lipid depots of triacylglycerols and cholesterol, decreased plasma lipid peroxides and body fat accumulation in Sprague-Dawley (SD) rats with high-fat induced NASH [20]. Dietary SPI reduced high-fat induced steatosis in the liver of SD rats [19], and decreased hepatic steatosis and diacylglycerols, changed microbiota populations, bile acid signaling and cholesterol homeostasis in Otsuka Long-Evans Tokushima fatty rats [54]. Feeding a diet containing soy protein concentrate enriched with isoflavones reduced fatty liver, and decreased plasma ALT, AST and triacylglycerol levels [21, 22], and increased activities of mitochondrial and peroxisomal *β*-oxidation, acetyl-CoA carboxylase, fatty acid synthase (FAS) and glycerol-3-phosphate acyltransferase in the liver of the obese Zucker rats [21]. Partial or full replacements of dietary casein by alcohol-washed SPI (devoid of isoflavones) or supplementation with soy isoflavones in the casein-based diet could effectively prevent the accumulation of lipid droplets in the liver of non-obese SD rats (Fig. 1) [55].

### Functional Components in the Soy

The bioactive components in soy that play major roles in mediating the hypolipidemic actions and improvement of NAFLD are not fully understood, and inconsistency exist in the literature. For example, soy protein markedly lowered serum triglycerides and cholesterol levels, and regulated gene expression involved in the synthesis of fatty acids in the liver of rats compared to casein protein. Supplementation of soy isoflavones had little effect on liver lipogenesis [56]. Simmen et al. [57] showed that suppression of fat droplet formation and accumulation in the liver of non-obese rats fed soy diets was independent of genistein [57]. Our study in SD rats also showed that intake of 20% alcohol-washed SPI with or without added isoflavones markedly lowered plasma triglycerides levels compared to a casein diet, however the added isoflavones had no additional effects [58].

*β*-conglycinin, one of the major storage proteins in soybean, was shown to reduce serum triglycerides, glucose and insulin levels [12], and prevented high-fat induced fatty liver in mice [59], and increased blood adiponectin level and insulin sensitivity in Wistar rats [23]. Our studies, using soy proteins with depletion of different subunits, further demonstrated that *α*’ subunit of *β*-conglycinin and all acidic polypeptides (A1 to A5) in glycinin were not required for the lipid-lowering effects and fatty liver reduction of soy proteins [60, 61]. This indicates that the other subunits of *β*-conglycinin and glycinin may play major roles in this regard.

Nevertheless, soy isoflavone extract markedly alleviated the high-fat induced hepatic steatosis and altered related gene expressions in an ovariectomized Wistar rat model for postmenopausal women [62]. Genistein and daidzein regulated hepatic lipogenesis, insulin resistance or adiposity and adipocytokines involved in hepatic steatosis [8, 63]. Administration of genistein reduced lipid accumulation in the livers and ameliorated fatty liver, improved insulin sensitivity, lipid profiles, liver injury, histological abnormalities and activated the antioxidant profiles, decreased the pre-inflammatory cytokines, IL-6 and TNF-*α*, and prevented oxidative damage in the high-fructose induced insulin-resistant rats [7]. Soy genistein and daidzein could inhibit oleic acid-induced intracellular lipid accumulation in human HepG2 liver cell lines [64]. In general, both soy protein and isoflavones appear to be effective in lowering liver and blood lipids, improving glucose tolerance and insulin sensitivity and reducing liver steatosis although some inconsistencies exist in the effects of isoflavones.

### Potential Molecular Mechanism(S) Involved

**Fig. 2:**
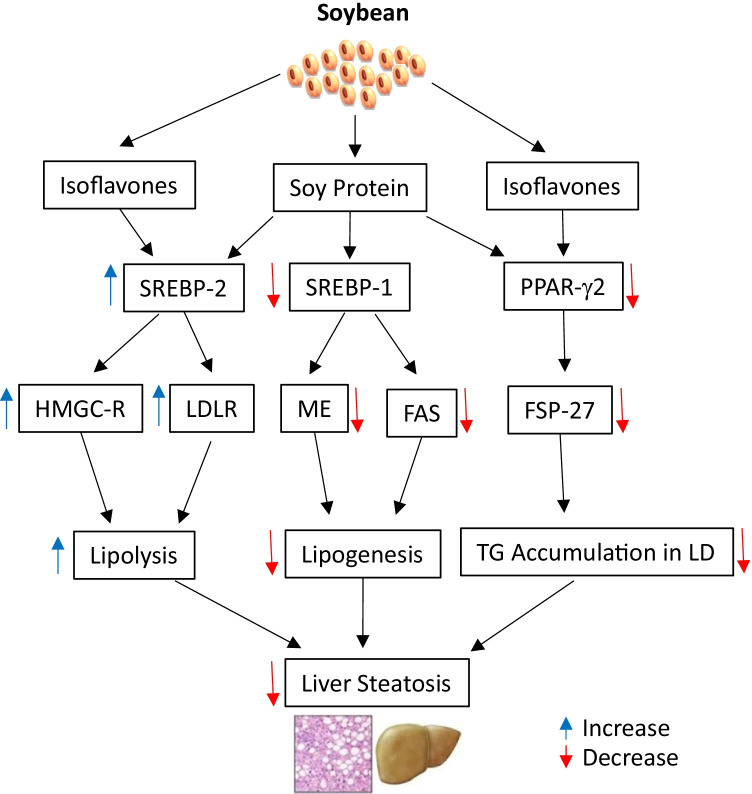
Potential molecular mechanism(s) involved in the soy effects on NAFLD in the non-obese models. Soy protein and isoflavones speed up hepatic lipolysis through activation of SREBP-2 and up-regulation of the downstream gene (HMGC-R and LDLR) expression, while soy protein suppresses hepatic lipogenesis by down-regulation of SREBP-1 and its target genes (such as ME and FAS). Moreover, both soy protein and isoflavones inhibit formation and accumulation of lipid droplets in liver via suppression of PPAR-*γ*2 and FSP-27 expression. FAS, fatty acid synthase; FSP-27, fat-specific protein 27; HMGC-R, 3-hydroxyl-3-methyl-glutaryl-CoA reductase; LD, lipid droplets; LDLR, low-density lipoprotein receptor; ME, malic enzyme; PPAR-*γ*2, peroxisome proliferation-activated receptor *γ*2; SREBP-1, sterol regulatory element-binding protein-1; TG, triglycerides

Although soy intake suppresses the formation and accumulation of liver lipid droplets and reduces triglyceride content in both obese [21, 22] and non-obese animal models [57], the mechanism(s) involved in the hypolipidemic actions and NAFLD prevention of soy are different in the two models. In non-obese rats (Fig. 2), dietary soy proteins down-regulated expression of hepatic genes for lipogenesis such as sterol regulatory element-binding protein-1 (SREBP-1), malic enzyme and FAS, while up-regulated expression of the genes for lipolysis such as SREBP-2, 3-hydroxy-3-methyl-glutaryl-CoA (HMGC) reductase, HMGC synthase and LDL receptor [65].

This is consistent with our results from proteomic analysis of liver proteins in the non-obese SD rats fed SPI or casein diets. Soy protein attenuated the abundance of the proteins involved in the lipogenesis and enhanced the proteins or enzymes in the lipolysis. In addition, both soy protein and supplemental isoflavones markedly reduced peroxisome proliferator-activated receptor-*γ*2 (PPAR*γ*2) and its target gene, fat-specific protein 27 (FSP27), in the liver, which was associated with decreased accumulation of hepatic lipid droplets [55]. FSP27 is a lipid droplet-associated protein, and promotes the formation of hepatic lipid droplets by enhancing triglyceride accumulation within lipid droplets and regulating fat storage [66]. Overexpression of FSP27 in the liver of the leptin-deficient (ob/ob) mice increased hepatic triglyceride content [67]. Our results suggested that prevention of hepatic lipid droplet accumulation by supplemented isoflavones was mainly mediated by suppression of hepatic FSP27 and that soy proteins reduced the abundance of FSP27 and hepatic triglyceride content, thereby preventing fatty liver [55]. This is in line with the effects of *β*-conglycinin that attenuated PPAR*γ*2 protein and FSP27 mRNA expression in mice [59].

However, in the obese rats, soy proteins enhanced hepatic lipogenesis and increased activities of hepatic FAS and plasma triacylglycerol levels [21], which might be due to increased blood insulin levels [68]. SPI could restore the suppressed *β*-catenin signaling pathway in the Zucker obese rats compared to their lean mates, and thereby attenuating hepatic fat accumulation, liver damage and hepatocellular vacuolation [69].

*β*-conglycinin is one of the most bioactive globulins in soy and modulated genes and proteins associated with hypolipidemic functions and preventive effects on NAFLD. *β*-conglycinin effectively prevented high-sucrose induced fatty liver through suppression of SREBP-1c and carbohydrate response element-binding protein mRNA [59], and lowered hepatic triglycerides, serum insulin and leptin concentrations and prevented high-fat induced fatty liver via suppression of liver PPAR*γ*2 and/or SREBP-1c protein in mice [59, 70]. *β*-conglycinin and *β*-conglycinin-derived peptides reduced liver weight and lipid content, and inhibited lipogenic gene expression and enzymatic activity and increased lipolytic enzyme activity in the rat models of NAFLD [71, 72]. It was proposed that the hypolipidemic effects of *β*-conglycinin might be due to increased insulin sensitivity of the liver, down-regulation of hepatic SREBP-1 [23] and PPARγ2 gene expressions [59], as well as acceleration of *β*-oxidation of fatty acids and suppression of FAS and/or increased triglycerides fecal excretion.

Hashidume et al. (2016) [73] showed that some of *β*-conglycinin effects were mediated through induction of hepatic fibroblast growth factor 21 (*FGF21)* expression and circulating FGF21 levels in a mouse model, which was regulated by activating transcription factor 4 (ATF4). It was further revealed that *β*-conglycinin ingestion resulted in methionine imbalance in portal blood as methionine content in *β*-conglycinin is only 1% compared to 2.2% in glycinin [74] and 3% in casein [73], which played a critical role in activation of the ATF4-FGF21 signaling axis and stimulation of the metabolic responses in hepatocytes [73]. The score of the sulfur-containing amino acids (the sum of methionine and cysteine) in *β*-conglycinin calculated against the requirement of rodents is 0.39, compared to 1.04 in casein (Table 3). Methionine is the first limiting amino acid in *β*-conglycinin for rodents. However, when the *β*-conglycinin diet was supplemented with enough methionine, the *β*-conglycinin effect on ATF4-FGF21 signaling was almost completely eliminated [73]. It was verified that all the other studies on *β*-conglycinin in rodents cited in this paper were supplemented with enough methionine or cysteine. Thus, methionine imbalance-induced mechanism might be not involved in the functions of *β*-conglycinin observed in those studies.

**Table 3:** Comparison of indispensable amino acid (IAA) content and ratios in casein and *β*-conglycinin

		Casein^b^		*β*-conglycinin^b^		*β*-conglycinin+Met^b,c^	
Indispensable Amino Acid (IAA)	IAA^a^ Req. (mg/g)	IAA (mg/g)	IAA ratio	IAA (mg/g)	IAA ratio	IAA (mg/g)	IAA ratio
Arginine	36	36	0.99	91	2.53	88.8	2.48
Histidine	26	30	1.16	21	0.82	20.6	0.80
Isoleucine	48	53	1.10	50	1.05	49.1	1.03
Leucine	86	92	1.06	80	0.93	78.5	0.91
Lysine	73	98	1.34	72	0.99	70.7	0.97
Met + Cys^d^	46	49	1.04	18	0.39	36.3	0.78
Phe^d^ + Tyr^d^	101	106	1.04	101	0.99	98.6	0.97
Threonine	38	40	1.07	25	0.67	24.5	0.65
Tryptophan	12	13	1.06	6	0.51	5.9	0.50
Valine	56	67	1.19	42	0.74	40.7	0.73

^a^Based on AIN-93G indispensable amino acid requirements for growing rodents

^b^IAA content were from Hashidume et al. [73]

^c,d^Met, methionine; Cys, cysteine; Phe, phenylalanine; Tyr, tyrosine

The other promising genes and proteins that may play important roles in mediating the reduction of liver steatosis by soy protein include hepatic Neuregulin 1 (NRG1), Erb-B2 Receptor Tyrosine Kinase 3 (ERBB3) and mitogen-activated protein kinase interacting serine/threonine kinase 1 (MKNK1). NRG1 and ERBB3 are membrane-bound proteins. ERBB3 could be modulated through phosphorylation by NRG1 to alleviate liver steatosis [75]. MKNK1 gene knocked-out mice were protected against a high-fat diet-induced obesity and detrimental effects such as impaired glucose tolerance, increased body weight gain and inflammatory biomarkers [76, 77]. A shotgun proteomics analysis showed that NRG1 and ERBB3 were the top activated proteins and MKNK1 was the top inhibited protein in the liver of the obese Zucker rats fed SPI [78]. This suggests that modulation of these molecules might be important cellular events by which soy protein exerts its hypolipidemic actions and alleviation of liver steatosis. However, this needs further investigation.

## Conclusions

Both soy protein and associated isoflavones have been shown to be hypolipidemic and play a role in reduction of liver steatosis and improving NAFLD-related syndrome in both human and animal studies. The molecular mechanism(s) involved are mainly through inhibiting lipogenesis by down-regulation of the transcription factors, SREBP-1c and PPARγ2, and their target genes, and enhancing lipolysis via up-regulation of SREBP-2 and its downstream genes in the non-obese models, while improving insulin resistance and restoring the suppressed *β*-catenin signaling pathway in the genetically obese models. The other benefits of soy components include protection of liver against oxidative damage and inflammation. Some effects of soy isoflavones and equol are sex-dependent, and the mechanism(s) involved remain unclear. Since most of the mechanism studies on soy actions were conducted in either cultured cells or rodent models that are known to differ in protein and indispensable amino acid requirements, whether the same mechanisms are shared in human remains to be determined. Overall, consumption of soy foods or supplements might be a useful strategy to mitigate the disease burden and prevalence of NAFLD, which is consistent with the new Canada’s Food Guide that recommends consumption of more plant-based protein foods [79].

## Data Availability

Not applicable.

## Abbreviations

ALT: Alanine aminotransferase
AST: Aspartate aminotransferase
ATF4: Activating transcription factor 4
BMI: Body mass index
ER: Estrogen receptor
ERBB3: Erb-B2 receptor tyrosine kinase 3
FAS: Fatty acid synthase
FGF21: Fibroblast growth factor 21
FSP27: Fat-specific protein 27
hs-CRP: High-sensitivity C-reactive protein
IAA: Indispensable amino acid
LDL: Low density lipoprotein
MDA: malondialdehyde
MKNK1: MAPK interacting serine/threonine kinase 1
NAFLD: Non-alcoholic fatty liver disease
NASH: Non-alcoholic steatohepatitis
NRG1: Neuregulin 1
PPARγ2: Peroxisome proliferator-activated receptor γ2
SD: Sprague-Dawley
SPI: Soy protein isolate
SREBP-1: Sterol regulatory element-binding protein-1
